# Identifying Metabolic Perturbations and Toxic Effects of *Rac*-Metalaxyl and Metalaxyl-M in Mice Using Integrative NMR and UPLC-MS/MS Based Metabolomics

**DOI:** 10.3390/ijms20215457

**Published:** 2019-11-01

**Authors:** Ping Zhang, Sheng Wang, Yuhan He, Yangyang Xu, Dongmei Shi, Furong Yang, Weizhong Yu, Wentao Zhu, Lin He

**Affiliations:** 1 Key Laboratory of Entomology and Pest Control Engineering, College of Plant Protection, Southwest University, Chongqing 400715, China; zpcauz@163.com (S.W.); hm20161027@163.com (Y.H.); zp8708@163.com (Y.X.); shidm48@163.com (D.S.); yfr200111@163.com (F.Y.); yuweizhong147@163.com (W.Y.); 2 Academy of Agricultural Sciences, Southwest University, Chongqing 400715, China; 3 State Cultivation Base of Crop Stress Biology for Southern Mountainous Land of Southwest University, Southwest University, Chongqing 400715, China; 4 Beijing Advanced Innovation Center for Food Nutrition and Human Health, Department of Applied Chemistry, China Agricultural University, Beijing 100193, China; pingz028@163.com

**Keywords:** metalaxyl, metabolomics, metabolic perturbation, amino acids, tryptophan metabolism, NMR, UPLC-MS/MS

## Abstract

Although metabolic perturbations are sensitive indicators for low-dose toxic effects, the metabolic mechanisms affected by *rac*-metalaxyl and metalaxyl-M in mammals from a metabolic profiling perspective remain unclear. In this study, the metabolic perturbations and toxic effects of *rac*-metalaxyl and metalaxyl-M in mice were carefully investigated using integrative nuclear magnetic resonance (NMR) and ultra-performance liquid chromatography-tandem mass spectrometry (UPLC-MS/MS) based metabolomics. Histopathology, NMR-based untargeted urine profile, multivariate pattern recognition, metabolite identification, pathway analysis, UPLC-MS/MS based targeted serum amino acids, and tryptophan pathway analysis were determined after *rac*-metalaxyl and metalaxyl-M exposure, individually. Histopathology indicated that metalaxyl-M induced greater hepatocellular inflammatory, necrosis, and vacuolation in mice than *rac*-metalaxyl at the same exposure dosage. The metabolic perturbations induced by *rac*-metalaxyl and metalaxyl-M were directly separated using partial least-squares discriminant analysis (PLS-DA). Furthermore, metabolite identification and pathway analysis indicated that *rac*-metalaxyl mainly induced ten urine metabolite changes and four pathway fluctuations. However, metalaxyl-M induced 19 urine metabolite changes and six pathway fluctuations. Serum amino acids and tryptophan pathway metabolite changes induced by *rac*-metalaxyl and metalaxyl-M were also different even at the same exposure level. Such results may provide specific insight into the metabolic perturbations and toxic effects of *rac*-metalaxyl and metalaxyl-M, and contribute to providing available data for health risk assessments of *rac*-metalaxyl and metalaxyl-M at a metabolomics level.

## 1. Introduction

Metalaxyl ((R,S)-methyl-2-(N-(2-methoxyacetyl)-2,6-dimethylanilino)propanoate, CAS 57837-19-1) is a widely used phenylamide fungicide in agriculture [1,2]. The fungicidal mechanisms of metalaxyl come from the specific inhibition of RNA polymerase-1 and uridine incorporation into RNA [3]. Metalaxyl is a chiral fungicide and consists of two enantiomers (*R*-enantiomer and *S*-enantiomer), which process similar physicochemical properties in non-chiral environment and different activities in chiral biological processes [4]. Previous study indicated that the fungicidal activity of metalaxyl almost entirely originated from its *R*-enantiomer [5]. To date, both racemate metalaxyl (*rac*-metalaxyl) and metalaxyl-M (consisting of 97.5% of *R*-enantiomer and 2.5% *S*-enantiomer) are used in agriculture (Figure S1). In some countries, *rac*-metalaxyl has been replaced by metalaxyl-M, which decreases application rate and reduces the potential side-effects of *rac*-metalaxyl on the environment and non-target organisms [6,7]. Studies indicated that metalaxyl could be detected in groundwater. Cytogenetic effects and cocarcinogenic potential of metalaxyl have also been verified in mice [8,9,10]. Therefore, a deeper knowledge of the differences in metabolic perturbations and toxic effects between *rac*-metalaxyl and metalaxyl-M at metabolomics level may provide comprehensive risk assessments for environment safety and human health.

Environmental metabolomics is defined as the application of metabolomics approaches to analyze the metabolic responses of the organism to environmental stimulis, including light, temperature, humidity, anoxic stress, environmental contaminants, etc. [11,12]. Environmental metabolomics have been widely applied in health risks and toxic effect assessments of environmental contaminants. In general, the concentration of these potentially harmful contaminants in the environment is often low, hence metabolomics would be more sensitive and useful in detecting these changes [13], compared to the indicators used in conventional toxicity tests, such as mortality, reproductive dysfunction, impaired growth, and aberrant behavior. Proton nuclear magnetic resonance (^1^H-NMR), gas chromatography-mass spectrometry (GC-MS) and liquid chromatography-mass spectrometry (LC-MS) are the main analytical platforms adopted in metabolomics studies, but ^1^H-NMR suffers from low sensitivity and may not detect metabolites with low abundance [14]. In recent years, the combinations of gas chromatography-quadrupole time of flight mass spectrometry (GC-Q-TOF/MS) and liquid chromatography-quadrupole time of flight mass spectrometry (LC-Q-TOF/MS) have been applied successfully in numerous metabolomics studies to achieve more sensitive and accurate metabolic profiling and screening of biomarkers [15]. For instance, Zhang et al. employed NMR-based metabolomics to study the toxic effects of municipal wastewater effluent in mice and found that municipal wastewater effluent induced amino acid, nucleotide, lipid, and energy metabolism perturbations, which result in hepatotoxicity and nephrotoxicity in mice [16]. Xu et al. adopted ^1^H-NMR based metabolomics to study the toxic effects of butachlor on gold fish and found that metabolic pathways related to oxidative stress, energy metabolism, amino acids metabolism, and neurotransmitter balance were significantly perturbed after butachlor exposure [17]. Gao et al. identified early urinary metabolic changes with long-term environmental cadmium exposure by LC-MS-based metabolomics and found N-methyl-l-histidine could be used as a potential biomarker for long-term cadmium exposure [18]. Thus, combining NMR and LC-MS-based metabolomics have the potential to further elucidate metabolic perturbations and toxic mechanisms of environmental pollutants with more reliability.

Hrelia et al. demonstrated the genotoxicity of metalaxyl on human and animal chromosomes in vitro [8]. Our previous researches revealed enantioselective metabolism of metalaxyl in rat hepatic microsomes [19,20] and metabolic pathway identifications in mice [21]. Recently, the effects of *rac*-metalaxyl and metalaxyl-M on earthworms have been evaluated by ^1^H-NMR based metabolomics [22]. Gu et al. compared the different metabolic profiles in adolescent rat induced by *R*-metalaxyl and *S*-metalaxyl, the two enantiomers of *rac*-metalaxyl using NMR-based serum metabolomics [23]. However, as two widely used commercial fungicides around the world, the differences in metabolic perturbations and toxic effects between *rac*-metalaxyl and metalaxyl-M in mammals were not clear, especially from the whole metabolic perturbations perspective depending on more sensitive and integrative metabolomics approaches.

The aim of this study was conducted to characterize the metabolic perturbations and toxic effects of *rac*-metalaxyl and metalaxyl-M in mice at molecular level using integrative nuclear magnetic resonance (NMR) and ultra-performance liquid chromatography-tandem mass spectrometry (UPLC-MS/MS) based metabolomics. Histopathology, NMR-based urine metabolomic profiling, multivariate pattern recognition, pathway analysis, UPLC-MS/MS based serum amino acids and tryptophan metabolic pathway analysis were carefully investigated. These results may provide specific insight into systematic assessment of metabolic perturbations and toxic effects of *rac*-metalaxyl and metalaxyl-M on mammals, and provide available data for health risk assessments of *rac*-metalaxyl and metalaxyl-M at perspective metabolomics level.

## 2. Results

### 2.1. Body Weight and Histopathology

**Figure 1:**
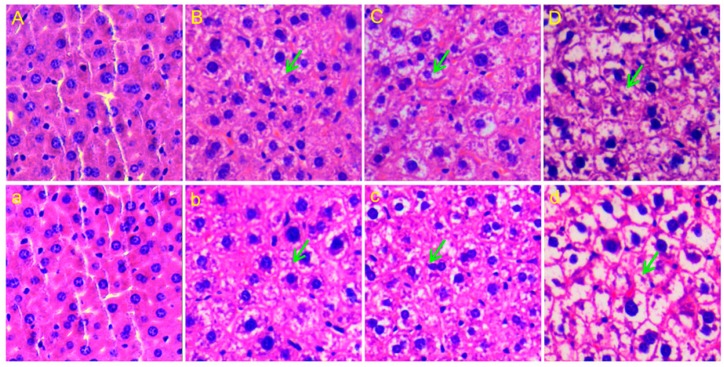
Representative histopathology sections of liver after treated with *rac*-metalaxyl: (**A**) control; (**B**) 10 mg/kg; (**C**) 30 mg/kg; (**D**) 60 mg/kg); and, metalaxyl-M: (**a**) control; (**b**) 10 mg/kg; (**c**) 30 mg/kg; (**d**) 60 mg/kg). The green arrows indicate hepatocellular necrosis and vacuolation.

After four weeks of exposure to *rac*-metalaxyl and metalaxyl-M, only one death was observed in the mice that received 60 mg/kg dosing. There were no significant changes in body weight observed between the *rac*-metalaxyl and metalaxyl-M treatment groups (Figure S2). Representative histopathology sections of liver after *rac*-metalaxyl and metalaxyl-M exposure are shown in Figure 1. Histopathological examinations showed that both *rac*-metalaxyl and metalaxyl-M induced hepatocellular inflammatory, necrosis, and vacuolation in mice liver even at lowest concentration (10 mg/kg). Furthermore, metalaxyl-M seemed to cause greater liver damage than *rac*-metalaxyl at the same dosage. These results clearly indicated that both *rac*-metalaxyl and metalaxyl-M could induce liver injury in mice.

### 2.2. Metabolomic Alterations

**Figure 2:**
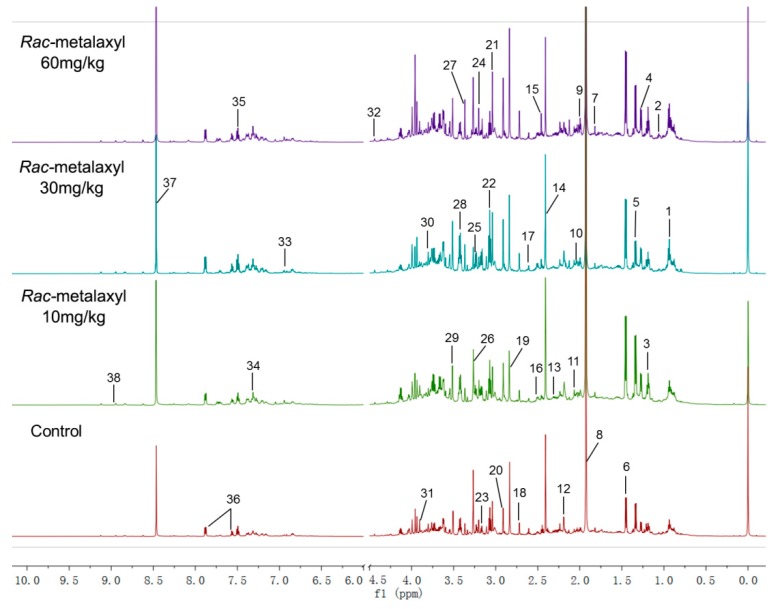
Representative ^1^H-NMR spectra of the urine sample after *rac*-metalaxyl exposure: 1, 3-Hydroxybutyrate; 2, Valine; 3, Methylmalonate; 4, Fucose; 5, Lactate; 6, Alanine; 7, N-Acetylglutamate; 8, Acetate; 9, Acetamide; 10, N-Acetylaspartate; 11, N-Actyl-glycoprotein; 12, Acetone; 13, Pyruvate; 14, Succinate; 15, α-Ketoglutarate; 16, Citrate; 17, Methylamine; 18, Dimethylamine; 19, Trimethylamine; 20, N,N-Dimethylglycine; 21, Creatine; 22, 3-Methylhistidine; 23, Choline; 24, Phosphorylcholine; 25, Glycerophosphocholine; 26, TMAO; 27, Scyllo-inositol; 28, para-Hydroxyphenylacetate; 29, Glycine; 30, Glycerol; 31, Guanidoacetate; 32, Trigonelline; 33, Aminohippurate; 34, Urocanate; 35, Benzoate; 36, Hippurate; 37, Formate; 38, Nicotinamide.

**Figure 3:**
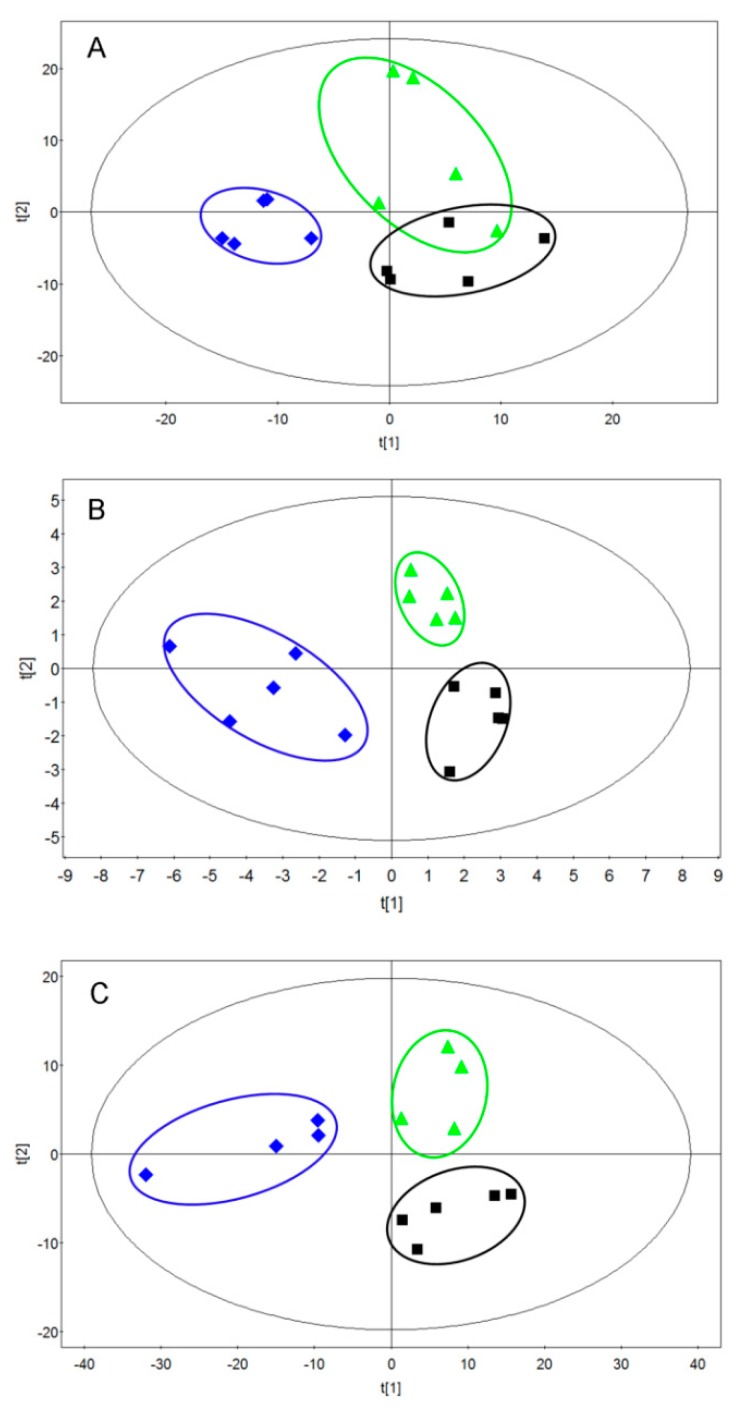
Partial least-squares discriminant analysis (PLS-DA) based on urine ^1^H-NMR spectra. (**A**) 10 mg/kg treatment (R2X = 0.729, R2Y = 0.762, Q2 = 0.823); (**B**) 30 mg/kg treatment (R2X = 0.773, R2Y = 0.846, Q2 = 0.917); (**C**) 60 mg/kg treatment (R2X = 0.807, R2Y = 0.854, Q2 = 0.891); (■) control group, (◆) metalaxyl-M treated group, (▲) *rac*-metalaxyl treated group.

In this study, metabolic alterations in urine were determined by ^1^H-NMR. In order to characterize the variation of metabolic profiles, partial least-squares discriminant analysis (PLS-DA) models were performed to analyze the obtained ^1^H-NMR data (Figure 2). The discriminations between the *rac*-metalaxyl, metalaxyl-M treated mice, and control group were calculated based on the first three components, and the results indicated that the *rac*-metalaxyl treated group, metalaxyl-M treated group, and control group were successfully separated with good R2X, R2Y, and Q2, which suggested all these models were applicable and had predictability values (Figure 3 and Figure S3). Such results demonstrated that both *rac*-metalaxyl and metalaxyl-M exposure obviously affected urine metabolic profile in mice.

^1^H-NMR spectra from both the metalaxyl-treated and the control group included rich information for endogenous metabolites. In this study, the resonances of those metabolites were comprehensively identified according to public databases and literature data [24,25,26]. A total of 38 urine metabolites were identified in mice urine (Table S1). Ten significantly changed metabolites (VIP > 1) were found between the *rac*-metalaxyl treated group and the control group. Moreover, a total of 19 metabolites were significantly changed between the metalaxyl-M treated group and the control group (Table 1). The pattern recognition showed that the metabolic profiles of urine altered with the increase of *rac*-metalaxyl and metalaxyl-M concentration, especially in high dose group (60 mg/kg). It was also found that there was a certain degree of dose–effect relationship between metabolites changes and metalaxyl dosage. For example, with the increasing of *rac*-metalaxyl and metalaxyl-M, the concentrations of alanine, citrate, and trimethylamine N-oxided (TMAO) increased, and the levels of methylamine (MA), trimethylamine (TMA), dimethylglycine (DMG), fucose, and hippurate decreased. In addition, the metabolic profile fluctuation degrees induced by metalaxyl-M seemed much larger than *rac*-metalaxyl.

### 2.3. Biological Pathway Changes

**Figure 4:**
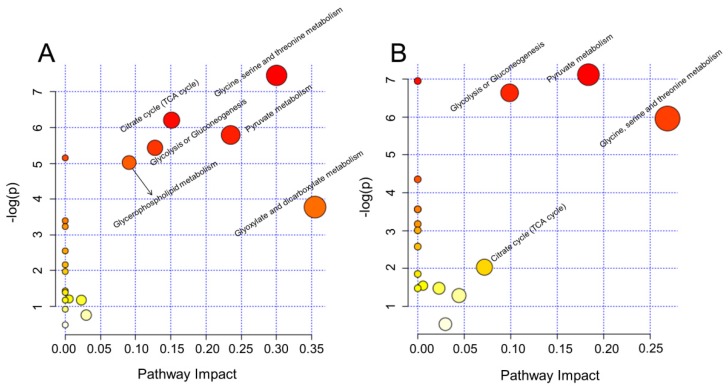
Summary of pathway analysis using MetaboAnalyst 4.0; (**A**) metalaxyl-M treatment group; (**B**) *rac*-metalaxyl treatment group.

Metabolomic profiling could not only reveal the alteration of individual metabolites but could also provide a comprehensive view of metabolic pathway changes induced by toxic exogenous compounds. In order to further characterize the metabolic variations, significantly changed metabolites were further analyzed using MetaboAnalyst 4.0 to reveal the major perturbed metabolic pathways induced by *rac*-metalaxyl and metalaxyl-M, individually. As a result, four metabolic pathways including glycine, serine and threonine metabolism, pyruvate metabolism, glycolysis or gluconeogenesis, and the citrate cycle (TCA cycle) were affected after *rac*-metalaxyl exposure. Moreover, a total of six metabolic pathways were fluctuated after metalaxyl-M exposure, including glyoxylate and dicarboxylate metabolism, glycine, serine, and threonine metabolism, pyruvate metabolism, citrate cycle (TCA cycle), glycolysis or gluconeogenesis and glycerophospholipid metabolism (Figure 4). Such pathways were highly related to lipid metabolism, energy metabolism, amino acid metabolism, and gut microbiota metabolism.

### 2.4. Serum Amino Acids Changes

**Figure 5:**
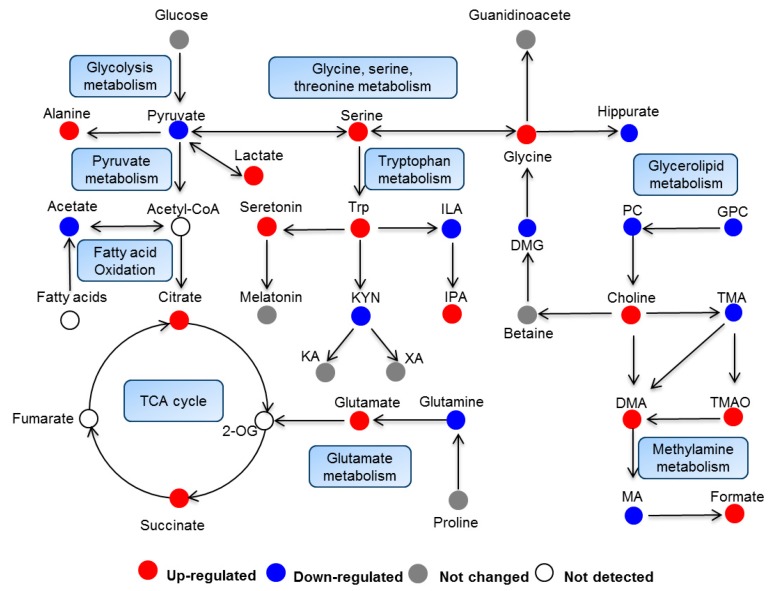
Perturbed pathways and changed metabolites induced by *rac*-metalaxyl and metalaxyl-M.

**Figure 6:**
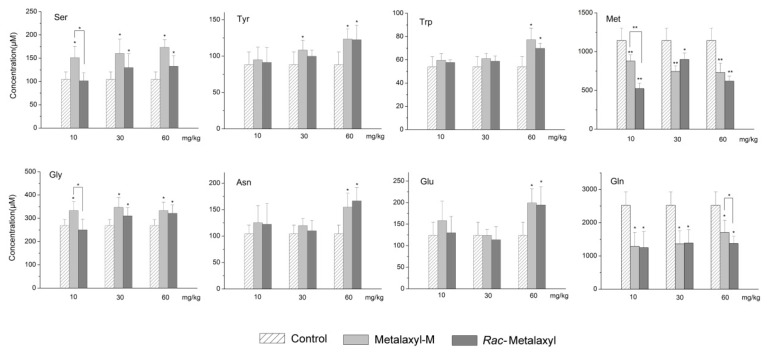
Alterations of serum amino acids induced by *rac*-metalaxyl and metalaxyl-M. Asterisks indicate significant differences (* *p* < 0.05 and ** *p* < 0.01) between treatment and control groups.

Amino acids played a vital role in the metabolic process of the organism. Moreover, the amino acids pathways were significantly changed in urine after *rac*-metalaxyl and metalaxyl-M exposure (Figure 5). Thus, 18 amino acids were precisely quantified in the serum using stable isotope-labeled internal standards by UPLC-MS/MS in the present study (Table S2 and Figures S4 and S5). As a result, eight amino acids were significantly changed after *rac*-metalaxyl and metalaxyl-M exposure, including the increased levels of serine (Ser), tyrosine (Tyr), tryptophan (Trp), glycine (Gly), asparagine (Asn), glutamate (Glu), and decreased levels of methionine (Met) and glutamine (Gln) (Figure 6).

### 2.5. Tryptophan Metabolites Changes

**Figure 7:**
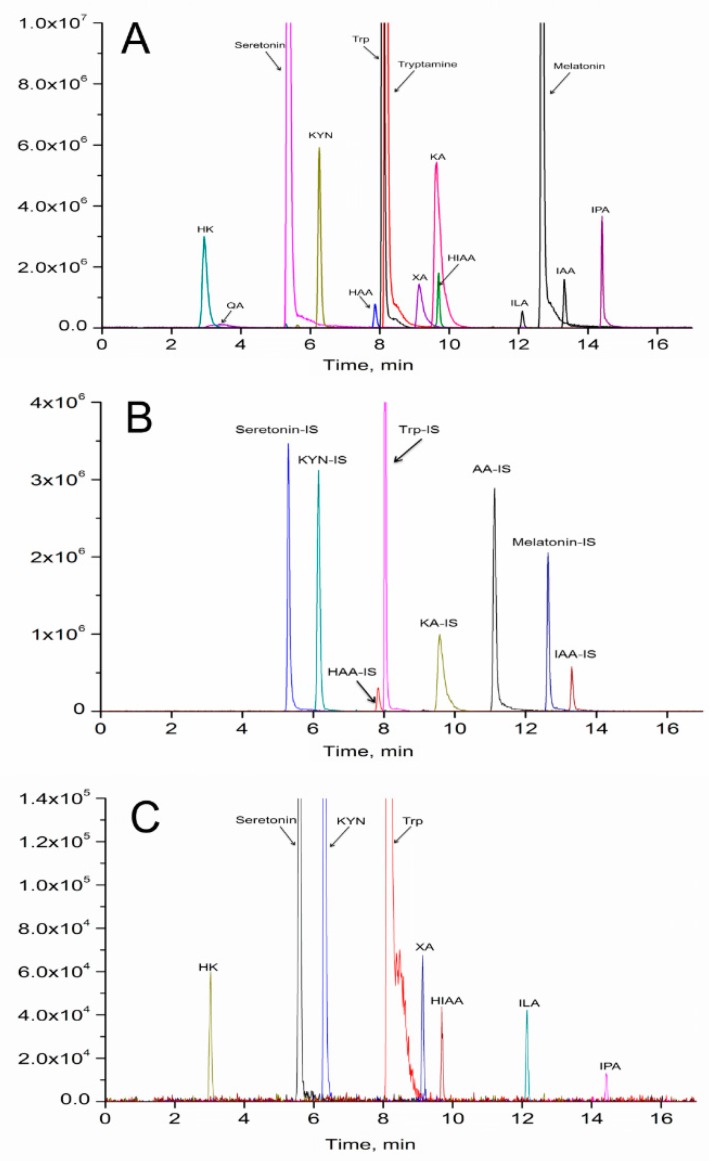
Representative UPLC-MS/MS chromatograms of tryptophan metabolites. (**A**) unlabeled tryptophan metabolites; (**B**) isotope labeled internal standards; (**C**) serum sample.

**Figure 8:**
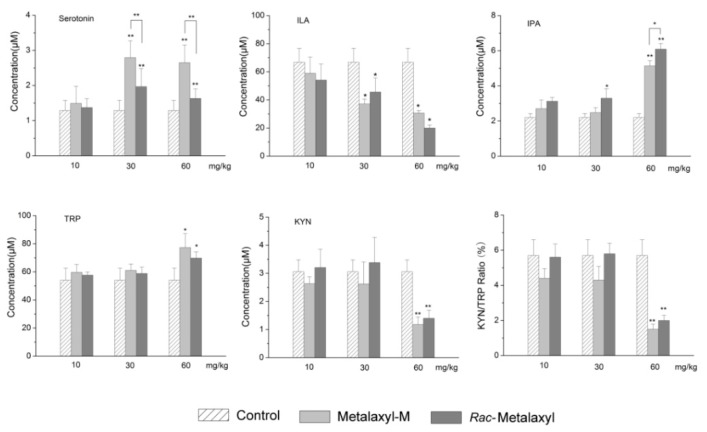
Tryptophan pathway metabolites changes induced by *rac*-metalaxyl and metalaxyl-M. Asterisks indicate significant differences (* *p* < 0.05 and ** *p* < 0.01) between treatment and control groups.

In our study, 15 metabolites in the tryptophan pathway were also precisely quantified in serum using stable isotope-labeled internal standards by UPLC-MS/MS (Table S3 and Figure 7). Only eight tryptophan metabolites were over the limit of quantitation (LOQ). *Rac*-metalaxyl and metalaxyl-M exposures induced the increased levels of serotonin and indole-3-propionic acid (IPA), and decreased levels of indole-3-lactic acid (ILA) and kynurenine (KYN). Furthermore, the KYN/Trp ratio decreased with the increase of *rac*-metalaxyl and metalaxyl-M dosage (Figure 8).

## 3. Discussion

Urine is a vital biofluid in mammals and could be easily collected in a noninvasive manner. Moreover, the metabolites components and concentrations in urine are good indicators of metabolic fluctuations. Comparison of metabolite profiles between the treatment group and the control group could obtain metabolic changes and reveal adverse effects mechanism of stressor. In this study, ^1^H-NMR and UPLC-MS/MS based metabolomics were employed to investigate the metabolic perturbations after *rac*-metalaxyl and metalaxyl-M exposure. The results showed that both *rac*-metalaxyl and metalaxyl-M induced systematic metabolic changes in urine and serum. *Rac*-metalaxyl and metalaxyl-M induced liver damages and metabolic pathway changes including glycine, serine, and threonine metabolism, citrate cycle (TCA cycle), pyruvate metabolism, glycolysis or gluconeogenesis, glycerophospholipid metabolism, and glyoxylate and dicarboxylate metabolism. These pathways are highly related to energy metabolism, lipid metabolism, amino acids metabolism, microbial metabolism, and tryptophan metabolism.

### 3.1. Body Weight Changes and Liver Damage

Only one mouse was dead in the 60 mg/kg metalaxyl-M treatment group during the whole experiment. After four weeks of exposure, no significant changes in body weight were observed between the control and treatment groups after *rac*-metalaxyl and metalaxyl-M exposure. Histopathology examination showed that *rac*-metalaxyl and metalaxyl-M can both induce hepatocellular inflammation, necrosis, and vacuolation in mice. Such results suggested *rac*-metalaxyl and metalaxyl-M exposure caused liver dysfunction in mice. Moreover, metalaxyl-M induced greater liver damage than *rac*-metalaxyl at the same dosage. The liver is a fundamental and essential organ for metabolism and detoxification of xenobiotics with abundant cytochrome P450 enzymes. The damages of the liver may greatly affect the detoxification of mice.

### 3.2. Energy Metabolism

Metabolites involved in energy metabolism were significant changed in mice urine after *rac*-metalaxyl and metalaxyl-M exposure, including citrate, succinate, lactate, alanine, pyruvate, and acetate. These metabolites are highly related to energy pathways, including the TCA cycle, pyruvate metabolism, and glycolysis or gluconeogenesis. Citrate and succinate are the main intermediates in the TCA cycle, which is mainly appeared in liver mitochondria. Although other factors cannot be excluded, the increased levels of citrate and succinate in the urine of metalaxyl-M-treated mice indicated that metalaxyl-M affected the activity of mitochondria enzymes involved in the TCA cycle, resulting in the enhancement of energy metabolism in treated mice. In addition, the decreased level of pyruvate and increased levels of lactate and alanine were highly related to pyruvate metabolism. Pyruvate could be converted to acetyl-coenzyme A (COA), which is a vital intermediate in the synthesis of citrate. Lactate and alanine can also be converted to pyruvate. Thus, the increased lactate and alanine could promote the level of acetyl-COA and then up-regulate the energy metabolism. To sum up, these results indicated the enhancement of energy metabolism in mice after *rac*-metalaxyl and metalaxyl-M exposure. Furthermore, the perturbation degrees induced by metalaxy-M were higher than *rac*-metalaxy in energy metabolism.

### 3.3. Lipid Metabolism

In this study, metalaxyl-M induced two lipid metabolism pathway fluctuations including glycerophospholipid metabolism, glyoxylate, and dicarboxylate metabolism. However, no lipid metabolism pathway was affected by *rac*-metalaxyl exposure. According to metabolomics data, metalaxyl-M induced the increased levels of choline and TMAO and decreased levels of PC and GPC. *Rac*-metalaxyl only induced the decreased level of GPC. Such metabolites were all related to lipid metabolism and had essential effect on cholesterol level in digestive system [27]. Choline is an important nutrient that is required for lipid transport, methyl-group metabolism, neurotransmitter synthesis, cell membrane structure, and signaling. In addition, choline is a constituent of cell membranes and lipoprotein phospholipids, which play an important role in the integrity of cell membrane and lipid metabolism. Increased choline levels in urine samples suggested the membrane fluidity was disrupted by metalaxyl-M. PC and GPC are known to be important endogenous metabolites related to maintenance of choline homeostasis and bile acids excretion. Moreover, PC, and GPC are not only essential components of cell membranes, but also protect cell from oxidative stress and lipotoxicity. The decreased levels of PC and GPC may be good indicators of oxidative stress and lipotoxicity induced by metalaxyl-M.

### 3.4. Gut Microbiota Metabolism

Hippurate is synthesized by the conjugation of glycine with benzoate in the liver of mammals, while benzoate is mainly derived from aromatic acid or plant phenolics by the action of intestinal microflora [28,29]. Thus, hippurate is the most widely detected urinary metabolites of host-microbial origin in rodents and humans and has become a vital biomarker for disease or gut microbial activity. A decreased level of hippurate in the urine at the 10 mg/kg dosage of the *rac*-metalaxyl and metalaxyl-M treatment groups reflected an alteration in gut microbiota. Furthermore the excretion of hippurate is used to assess liver function. Thus, the decreased hippurate in urine also indicates the liver dysfunction caused by *rac*-metalaxyl and metalaxyl-M exposure, which is accordance with our previous histopathology study. Another example of demonstrating the perturbation of the microbiota metabolism is methylamine metabolism. Methylamines can be derived from dietary choline, which break down to MA, DMA, and TMA by gut microflora [30]. As a consequence, the increased levels of choline and DMA, and the decreased level of TMA clearly indicated the fluctuations in gut microbiota induced by *rac*-metalaxyl and metalxyl-M. In addition, metalxyl-M induced higher gut microbiota metabolism fluctuations than *rac*-metalaxyl even at the same dosage.

### 3.5. Amino Acids Metabolism

Amino acids play important roles in the growth and development of organisms [31,32]. Growing evidence shows that some amino acids are vital regulators of key metabolic pathways that are necessary for maintenance, growth, development, reproduction, and immunity in organisms [33,34]. In this study, urine metabolomics data revealed that the glycine, serine, and threonine metabolism pathways were significantly changed due to *rac*-metalaxyl and metalaxyl-M exposure. Normal amino acids metabolism is essential for liver function to regulate nonessential amino acid synthesis, interconversion of protein, carbohydrates, and lipids, ammonia metabolism, and oxidation for energy [35,36,37]. Evidence showed that glycine, serine, and threonine metabolism pathways played vital roles in cancer metabolism [38]. Abnormal metabolism of amino acids may disturb whole body homeostasis, impair growth and development, and even cause death [39,40]. UPLC-MS/MS targeted serum amino acids analysis indicated increased levels of serine, glutamate, tyrosine, glycine, asparagine, and tryptophan, and decreased levels of methionine and glutamine in response to the *rac*-metalaxyl and metalxyl-M treatment. Furthermore, the degrees of some amino acids fluctuations induced by metalaxyl-M were higher than *rac*-metalaxyl at the same dosage, which indicates metalaxyl-M has greater effect on amino acids metabolism than *rac*-metalaxyl. Some amino acids serve as essential precursors for the synthesis of low-molecular-weight hormones. For example, tyrosine is the precursor for the synthesis of dopamine, norepinephrine, epinephrine and thyroid hormones. Some amino acids directly participate in cell signaling, cell metabolism, and oxidative stress [41,42,43]. Glutamate and aspartate mediate the transfer of reducing equivalents across the mitochondrial membrane and thus regulate glycolysis and cellular redox state [44]. Glycine, serine, and methionine actively participate in one-carbon metabolism and the methylation of protein and DNA, therefore regulating gene expression and biological activity of protein [34].

### 3.6. Tryptophan Metabolism

Tryptophan is an essential amino acid required for protein synthesis and could be converted to many biologically active compounds including KYN, serotonin, AA, NA, and tryptamine. Tryptophan is mainly metabolized through serotonin, indole, and kynurenine pathways. The former two pathways do not involve the cleavage of the indole ring and kynurenine pathway involves the cleavage of the indole ring. In the present study, 15 metabolites in the tryptophan metabolic pathway were accurately quantified based on isotope-stable internal standards in serum after *rac*-metalaxyl and metalaxyl-M exposure. The increased levels of IPA, tryptophan, serotonin and decreased levels of KYN and ILA indicated that the tryptophan pathway was significantly perturbed in mice after *rac*-metalaxyl and metalxyl-M exposure. More than 95% of free tryptophan is metabolized through KYN pathway. Growing studies found that perturbations in the levels of the KYN pathway have been related to the pathogenesis of many diseases, including cancer, neurodegenerative disease, age-related disease, etc. The first and rate-limiting step in KYN pathway is converting tryptophan to KYN, which is catalyzed by IDO and TDO in mammals. TDO mainly exists in the liver and its expression is induced by tryptophan concentration or corticosteroids. IDO is founded in most mammalian cells, which is induced by the proinflammatory cytokine interferon (IFN-γ) and other immune stimulants. Thus, TDO affects systemic tryptophan levels by regulating tryptophan concentration in the blood, while IDO acts locally to control tryptophan levels in response to inflammation. Generally, the KYN/Trp ratio is employed to evaluate IDO and TDO activity. The decreasing trend of KYN/Trp ratio indicated the activities of IDO and TDO were depressed with the increased dosage. Serotonin is also a main pathway for tryptophan metabolism, which occurs in distinct cell types in mammal. The main function of serotonin is known to regulate animal behaviors including sleep, mood, appetite, circadian rhythm, and reproduction. Considering its function, the increased level of serotonin suggested the mice behaviors were affected in response to *rac*-metalaxyl and metalaxyl-M. Together, these results showed that the tryptophan metabolism was sensitive to *rac*-metalaxyl and metalaxyl-M exposure in mice. Such exposures can perturb the growth, development, behaviors and immunity in mice.

## 4. Materials and Methods

### 4.1. Chemicals and Materials

*Rac*-metalaxyl (purity ≥ 98.5%) and metalaxyl-M (purity ≥ 98.0%) were obtained from Institute for Control of Agrochemicals, Ministry of Agriculture and Rural Affairs (Beijing, China). Deuterium oxide (D_2_O, 99.9% D) and sodium 3-trimethylsilyl [2,2,3,3-^2^H_4_] propionate (TSP) were bought from Cambridge Isotope Laboratories, Inc. (Tewksbury, MA, USA). Unlabled tryptophan, quinolinic acid (QA), xanthurenic acid (XA), serotonin hydrochloride, indole-3-acetic acid (IAA), tryptamine, indole-3-propionic acid (IPA), 3-hydroxyanthranilic acid (HAA), 5-hydroxyindole-3-acetic acid (HIAA), 3-hydroxykynurenine (HK), kynurenine (KYN), kynurenic acid (KA), DL-indole-3-lactic acid (ILA), melatonin, unlabeled amino acids, and [U-^13^C, U-^15^N] labeled cell free amino acid mixture were purchased from Sigma-Aldrich (St. Louis, MO, USA). ^2^H_5_-tryptophan, ^2^H_2_-tryptamin, ^2^H_4_-melatonin, ^2^H_2_-IAA, ^2^H_2_-HIAA, ^2^H_5_-KA, and ^2^H_4_-serotonin were purchased from C/D/N Isotopes Inc. (Pointe-Claire, QC, Canada). ^2^H_4_-KYN and ^2^H_2_-HAA were obtained from Buchem (Apeldoorn, Netherlands). K_2_HPO_4_·3H_2_O, and NaH_2_PO_4_·2H_2_O were bought from Sinopharm Chemical Co., Ltd. (Beijing, China). Solvents for sample preparation and UPLC-MS/MS analysis were HPLC grade and purchased from Merck Chemicals (Shanghai, China). Ultrapure water was generated by a Milli-Q system from Millipore (Billerica, MA, USA).

### 4.2. Animals and Animal Treatments

Eight-week-old male mice (*Mus musculus*, ICR) were purchased from the Vital River Laboratory Animal Company (Beijing, China) and housed at 25 ± 3 °C, with 50 ± 5% relative humidity, and a 12/12 h light/dark cycle, with free access to water and food. After two weeks of acclimatization, a total of 35 mice were randomly assigned to seven groups with five mice in each group, including a control group, three *rac*-metalaxyl treated groups, and three metalaxyl-M treated groups, and transferred to metabolic cages. Based on the LD_50_ (667 mg/kg) of metalaxyl reported by Environmental Protection Agency (USA), we adopted doses of 10, 30 and 60 mg/kg for *rac*-metalaxyl and metalaxyl-M treated groups. *Rac*-metalaxyl and metalaxyl-M were suspended in corn oil and given to mice by gavage daily for 28 consecutive days. The volume of administrated corn oil was calculated based on the weight of mice at 10 mL/kg and the control group was treated with an equivalent volume of corn oil. Urine samples were collected every 24 h and body weights were also recorded daily. At the end of feeding trails, blood was collected into Na-heparin tubes from the eyes, and plasma samples were obtained after centrifugation. All urine and plasma samples were stored at −80 °C before detection. Sections of liver were taken and stored in formalin solution for histological assessments. All animal experiments were performed in accordance with the current Chinese legislation and approved by the Institutional Animal Care and Use Committee of China Agricultural University (Approval Number CAU-2016-1003-05; approval date: 03/10/2016).

### 4.3. Histopathology

The liver tissues were fixed in 10% formalin for at least 12 h and cut into 4 μm paraffin sections. Liver slides were stained with hematoxylin and eosin (H&E) and observed under a microscope. Microscope assessments were conducted as a paid service by a qualified pathologist.

### 4.4. Metabolomic Profilings

400 μL of urine and 200 μL phosphate sodium buffer (0.2 M NaH_2_PO_4_ and 0.2 M Na_2_HPO_4_, PH = 7.4) were mixed to minimize variations in the pH of the urine. The mixture was homogenized and centrifuged at 10,000 rpm for 10 min at 4 °C to remove any precipitates. 500 μL aliquots of supernatant from each urine sample was added with 50 μL TSP/D_2_O (3 mM final concentration) and transferred into 5 mm NMR tubes for analysis. The TSP acted as a chemical shift reference (δ = 0.00 ppm) and the D_2_O provided a lock signal. Water signals were suppressed by presaturation.

A Bruker AV600 Spectrometer (Bruker CO., Germany) was adopted to acquire ^1^H-NMR spectra of all urine samples at 298K, the spectrum was acquired with a standard pulse sequence (nuclear overhauser enhancement spectroscopy (NOESY)) using 64 free induction decay (FIDs), 64k data points. The FIDs were weighted by an exponential function with a 0.3 Hz line-broadening factor prior to Fourier transformation. After Fourier transformation, the phase and baseline of the spectra were manually corrected using MestReNova 9.1.0 from Mestrelab Research (Santiago DE Compostela, Spain). All the spectra were referenced to TSP (δ = 0.00 ppm). Each spectrum was segmented into 0.02 ppm chemical shift bins corresponding to the range from 0.30 to 10 ppm. The region at 4.50–6.00 ppm was excluded to remove the effects of imperfect water suppression efficiency and urea signal. Then, all remaining regions were scaled to the total integrated area of the spectra to facilitate comparison among the samples.

Multivariate data analysis was performed with the software package SIMCA-P 11.0 (Umetrics, Sweden). Partial least-squares discriminant analysis (PLS-DA) was used to explore the main effects in the NMR data set. All the metabolites were identified according to public databases and previous studies [24,25,26]. Significantly changed metabolites were identified on one-way ANOVA and the variable importance in projection score (VIP > 1).

### 4.5. UPLC-MS/MS Analysis

The UPLC-MS/MS system consisted of an Agilent 1290 UPLC system (Agilent Technologies, Santa Clara, CA, USA) and a QTrap 5500 tandem mass spectrometer (AB Sciex, Toronto, ON, Canada). The instrument equipped with electrospray ionization (ESI) source and operated using Analyst 1.5.2 software (AB Sciex, Toronto, ON, Canada). Multiple reaction monitoring (MRM) and positive ion mode were used for detection (Tables S4 and S5). A Phenomenex EZfaast C18 column (250 mm × 2.0 mm, 4 μm) and a Waters Atlantis T3 column (150 mm × 2.1 mm, 3 μm) were adopted to determine amino acids and tryptophan metabolites separately with mobile phase A (0.1% formic acid in water, *v*/*v*) and B (0.1% formic acid in acetonitrile, *v*/*v*). The gradient was shown in Tables S6 and S7. The sample preparation and detection method were adopted according to the previous study [45,46].

### 4.6. Biological Pathway Analysis

Typically, an integrative analysis based on the significantly changed metabolites was performed to explain the major perturbed biological pathways induced by *rac*-metalaxyl and metalaxyl-M exposure. In this study, metabolic pathway perturbations were conducted by MetaboAnalyst 4.0 (http://www.metaboanalyst.ca/) and related metabolic pathway profiles were determined based on Kyoto Encyclopedia of Genes and Genomes (KEGG) pathway database (http://www.kegg.jp/kegg/pathway.html).

### 4.7. Statistical Analysis

One-way analysis of variance (ANOVA) was used to evaluate the statistical differences of biological parameters between treated groups and control group. All analyses were performed using SPSS 13.0 software (SPSS Inc., Chicago, IL, USA). A value of *p* < 0.05 was considered significant.

## 5. Conclusions

In this study, the metabolic perturbations and toxic effects of *rac*-metalaxyl and metalaxyl-M in mice were carefully investigated using integrative NMR and UPLC-MS/MS based metabolomics. Histopathology, untargeted urine profile, multivariate pattern recognition, pathway analysis, targeted serum amino acids, and tryptophan pathway analysis were determined after *rac*-metalxyl and metalaxyl-M exposures, individually. The results indicated that metalaxyl-M induced greater hepatocellular inflammatory, necrosis, and vacuolation in mice than *rac*-metalaxyl even at the same exposure dosage. The metabolic perturbations induced by *rac*-metalaxyl and metalaxyl-M were directly separated according to partial least-squares discriminant analysis (PLS-DA). Furthermore, metabolite identification and pathway analysis indicated that *rac*-metalaxyl mainly induced ten urine metabolites changes and four pathways fluctuations including glycine, serine, and threonine metabolism, pyruvate metabolism, glycolysis or gluconeogenesis, and TCA cycle. However, metalaxyl-M induced 19 urine metabolites changes and six pathways fluctuations including glyoxylate and dicarboxylate metabolism, glycine, serine, and threonine metabolism, pyruvate metabolism, TCA cycle, glycolysis or gluconeogenesis, and glycerophospholipid metabolism. Serum amino acids detection showed that eight amino acids were significantly changed after *rac*-metalaxyl and metalaxyl-M exposure, including the increased levels of serine, tyrosine, tryptophan, glycine, asparagine, and glutamate, and decreased levels of methionine and glutamine. Tryptophan pathway analysis indicated *rac*-metalaxyl and metalaxyl-M exposures induced the increased levels of serotonin and IPA, and decreased levels of ILA and KYN. Such results provide specific insight into systematic assessments of the metabolic perturbations and toxic effects of *rac*-metalaxyl and metalaxyl-M on mammals, and provide available data for health risk assessments of *rac*-metalaxyl and metalaxyl-M at perspective metabolomics level.

## Figures and Tables

**Table 1:** Alterations of urine metabolites induced by *rac*-metalaxyl and metalaxyl-M in mice.

No.	Metabolites	HMDB ID	Chemical Shift (ppm)	VIP	Fold Change (MX)			Trend	Fold Change (R-MX)			Trend
					10 mg/kg	30 mg/kg	60 mg/kg		10 mg/kg	30 mg/kg	60 mg/kg	
1	Fucose	HMDB0000174	1.25 (d, CH_3_); 3.77 (m, CH);3.81 (m, CH); 5.21 (d, CH)	1.07	0.91	0.96	0.99	-	0.94	0.76 *	0.68 *	↓
2	Lactate	HMDB0000190	1.33 (d, CH_3_); 4.11 (q, CH)	2.81	1.22 *	0.96	1.09	↑	1.30 *	1.63 **	1.19 *	↑
3	Alanine	HMDB0000161	1.46 (d, CH_3_); 3.78 (q, CH)	3.85	1.09	1.14 *	1.22 *	↑	1.20 *	1.04	0.91	↑
4	Acetate	HMDB0000042	1.92 (s, CH_3_)	6.54	0.86	0.85	0.96	-	0.94	0.86	0.52 *	↓
5	Pyruvate	HMDB0000243	2.37 (s, CH_3_)	1.26	0.91	0.99	0.81 *	↓	0.77 *	0.71 *	0.69 **	↓
6	Succinate	HMDB0000254	2.41 (s, CH)	2.28	1.19	1.09	1.07	-	1.41 *	1.34 *	1.36 *	↑
7	Citrate	HMDB0000094	2.54 (d, CH_2_), 2.69 (d, CH′_2_)	1.30	1.23	1.15	1.13	-	0.93	1.13	1.57 *	↑
8	MA	HMDB0000164	2.61 (s, CH_3_)	1.22	0.90	0.87 *	0.79 **	↓	0.8 **	0.81 **	0.77 **	↓
9	DMA	HMDB0000087	2.72 (s, CH_3_)	1.15	1.35 *	1.02	1.07	↑	1.36 **	1.24 **	1.46 **	↑
10	TMA	HMDB0000906	2.87 (s, CH_3_)	1.74	0.96	0.92	0.84 *	↓	1.00	0.73 *	0.87 *	↓
11	DMG	HMDB0000092	2.93 (s, CH_3_); 3.73 (s, CH_2_)	1.43	0.91	1.03	1.10	-	0.96	0.83 *	0.68 *	↓
12	Choline	HMDB0000097	3.20 (s, CH_3_); 3.52 (m, N-CH_2_); 4.07 (m, O-CH_2_)	2.45	1.12	1.05	1.12	-	1.11	1.58 **	1.49 **	↑
13	PC	HMDB0001565	3.22 (s, CH_3_); 3.59 (m, N-CH_2_);4.17 (m, O-CH_2_)	1.40	0.98	0.88	0.93	-	0.62 **	0.82	0.68 **	↓
14	GPC	HMDB0000086	3.23 (s, CH_3_); 3.68 (m, N-CH_2_); 3.69 (m, O-CH_2_); 4.33 (m, P-O-CH_2_)	2.26	0.85 *	0.82 *	0.94	↓	0.70	0.71 *	0.61 **	↓
15	TMAO	HMDB0000925	3.27 (s, CH_3_)	2.99	1.30	0.93	0.81	-	1.19	1.13	1.61 *	↑
16	Scyllo-inositol	HMDB0006088	3.35 (s, CH)	2.17	1.15 **	1.34 **	1.36 **	↑	1.52 **	1.79 **	2.00 **	↑
17	Glycine	HMDB0000123	3.56 (s, CH_2_)	2.46	1.38 *	1.18 **	1.01	↑	1.39 **	1.10	1.26 *	↑
18	Hippurate	HMDB0000714	3.97 (d, CH_2_); 7.55 (t, CH); 7.64 (t, CH); 7.84 (d, CH)	1.72	0.84 *	0.96	1.01	↓	0.86	0.85 *	0.78 *	↓
19	Formate	HMDB0000142	8.46 (s, CH)	2.94	1.08	1.13	1.01	-	1.44 **	1.16	1.55 **	↑

* *p* < 0.05, ** *p* < 0.01 (one-way ANOVA). MA, methylamine; DMA, dimethylamine; TMA, trimethylamine; DMG, dimethylglycine; PC, phosphorylcholine; GPC, glycerophosphocholine; TMAO, trimethylamine N-oxided.

## Supplementary Materials

Supplementary materials can be found at https://www.mdpi.com/1422-0067/20/21/5457/s1.

## Abbreviations

UPLC-MS/MS	Ultra performance liquid chromatography-tandem mass spectrometry
NMR	Nuclear magnetic resonance
PLS-DA	Partial least-squares discriminant analysis
LOQ	Limit of quantitation
TCA	Tricarboxylic acid
COA	Acetyl-coenzyme A
MA	Methylamine
DMA	Dimethylamine
TMA	Trimethylamine
DMG	Dimethylglycine
PC	Phosphorylcholine
GPC	Glycerophosphocholine
TMAO	Trimethylamine N-oxided
