# Supplementary material for: Identifying Metabolic Perturbations and Toxic Effects of Rac-Metalaxyl and Metalaxyl-M in Mice Using Integrative NMR and UPLC-MS/MS Based Metabolomics
Source: Int J Mol Sci. 2019 Nov 1;20(21):5457. doi: 10.3390/ijms20215457 (PMC6862379; doi:10.3390/ijms20215457)
Supplement: Supplementary file 1 [file ijms-20-05457-s001.pdf]

Supplementary Material

Identifying metabolic perturbations and toxic effects of *rac*-metalaxyl and metalaxyl-M in mice using integrative NMR and UPLC-MS/MS based metabolomics

Ping Zhang[1,2,3,*], Sheng Wang[1,2], Yuhan He[1,2,3], Yangyang Xu[1,2], Dongmei Shi[1,2], Furong Yang[1,2], Weizhong Yu[1,2], Wentao Zhu[4] and Lin He [1,2,3,*]

[1] Key Laboratory of Entomology and Pest Control Engineering, College of Plant Protection, Southwest University, Chongqing 400715, P.R.China

[2] Academy of Agricultural Sciences, Southwest University, Chongqing 400715, P.R.China

[3] State Cultivation Base of Crop Stress Biology for Southern Mountainous Land of Southwest University, Southwest University, Chongqing 400715, P.R.China

[4] Beijing Advanced Innovation Center for Food Nutrition and Human Health, Department of Applied Chemistry, China Agricultural University, Beijing 100193, P.R.China.

Page S-3: Table S1. ^{1}H-NMR chemical shifts for metabolites assigned in urine samples

Page S-4: Table S2. The calibration curves and the LOQ of amino acids

Page S-5: Table S3. The calibration curves and the LOQ of the tryptophan metabolites

Page S-6: Table S4. List of MRM parameters of amino acids and their internal standards

Page S-7: Table S5. List of MRM parameters of tryptophan metabolites and its internal standards

Page S-8: Table S6. The gradient of UPLC-MS/MS for amino acids separation

Page S-9: Table S7. The gradient of UPLC-MS/MS for tryptophan metabolites separation

Page S-10: Figure S1. Chemical structures of *rac*-metalaxyl and metalaxyl-M

Page S-11: Figure S2. Body weight changes of mice after *rac*-metalaxyl and metalaxyl-M exposure.

Page S-12: Figure S3. Partial least-squares discriminant analysis (PLS-DA) based on urine ^{1}H-NMR spectra. (A) metalaxyl-M (R2X = 0.724, R2Y = 0.857, Q2 = 0.932); (B) *rac*-metalaxyl(R2X = 0.615, R2Y = 0.71, Q2 = 0.824); (■) control group, (▲) 30 mg/kg treated group (◆) 60 mg/kg treated group

Page S-13: Figure S4. Representative UPLC-MS/MS chromatograms of alanine, valine and their stable isotope labeled internal standards.

Page S-14: Figure S5. Representative LC-MS/MS chromatograms of amino acids. (A) unlabeled amino acids metabolites; (B) isotope labeled internal standards; (C) serum sample

Table S1 ^{1}H-NMR chemical shifts for metabolites assigned in urine samples

Key	Metabolites	Chemical Shift (ppm)
1	3-Hydroxybutyrate	1.20(d,CH3); 2.31(m, α-CH); 2.41(m, α-CH'); 4.16(m, CH)
2	Valine	0.99(d, γ-CH3); 1.04(d, γ-CH3')
3	Methylmalonate	1.24(d,CH3)
4	Fucose	1.25(d, CH3); 3.77(m, CH);3.81(m, CH); 5.21(d, CH)
5	Lactate	1.33(d, CH3); 4.11(q, CH)
6	Alanine	1.46(d, CH3); 3.78(q, CH)
7	N-Acetylglutamate	1.86(m,β-CH); 2.04(s,CH3); 2.06(m,β-CH'); 2.25(t, γ-CH2)
8	Acetate	1.92(s,CH3)
9	Acetamide	1.99(s,CH3)
10	N-Acetylaspartate	2.02(s,VH3); 2.50(m,α-CH); 2.70(m,α-CH'); 4.39(m,β-CH);7.92(d,)
11	N-Actyl-glycoprotein	2.04(s,CH3)
12	Acetone	2.23(s,CH3)
13	Pyruvate	2.37(s,CH3)
14	Succinate	2.41(s,CH)
15	α-Ketoglutarate	2.45(t,γ-CH2); 3.01(t,β-CH2)
16	Citrate	2.54(d,CH2), 2.69(d,CH'2)
17	Methylamine	2.61(s,CH3)
18	Dimethylamine	2.72(s, CH3)
19	Trimethylamine	2.87(s,CH3)
20	N,N-Dimethylglycine	2.93(s,CH3); 3.73(s,CH2)
21	Creatine	3.04(s,CH3); 3.93(s,XH2)
22	3-Methylhistidine	3.09(m,β-CH), 3.20(m,β-CH'), 3.71(sCH3,), 3.93(m,α-CH),7.04(s,H2), 7.68(s,H4)
23	Choline	3.20(s,CH3); 3.52(m,N-CH2); 4.07(m,O-CH2)
24	Phosphorylcholine	3.22(s, CH3); 3.59(m, N-CH2);4.17(m, O-CH2)
25	Glycerophosphocholine	3.23(s, CH3); 3.68(m, N-CH2); 3.69(m, O-CH2); 4.33(m, P-O-CH2)
26	TMAO	3.27(s,CH3)
27	Scyllo-inositol	3.35(s, CH)
28	para-Hydroxyphenylacetate	3.45(s,CH2); 6.87(d,CH(2,6)); 7.17(d,CH(3,5))
29	Glycine	3.56(s,CH2)
30	Glycerol	3.57(m,CH2); 3.62(m,CH2'); 3.79(m,CH)
31	Guanidoacetate	3.80(s,CH2)
32	Trigonelline	4.44(s,CH3); 8.08(t,CH(4)); 8.84(dd,CH(3,5)); 9.13(s,CH(1))
33	Aminohippurate	6.86(d,CH(2,6)); 7.70(d,CH(3,5))
34	Urocanate	6.39(d,α-CH); 7.28(d,β-CH); 7.37(s,C-CH-NH); 7.82(s,N-CH=NH)
35	Benzoate	7.49(t,CH(3,5)); 7.54(t,CH(4)); 7.88(m,CH(2,6))
36	Hippurate	3.97(d,CH2); 7.55(t,CH(3,5)); 7.64(t,CH(4)); 7.84(d,CH(2,6))
37	Formate	8.46(s,CH)
38	Nicotinamide	7.59(dd,CH(5)); 8.24(dd,CH(4)); 8.72(dd,CH(4)); 8.94(s,CH(2))

Table S2 The calibration curves and the LOQ of amino acids

Compound	Internal standard	Linearity	R^2	Linear Range (μmol/L)	LOQ (μmol/L)
Gln	Gln-IS	y = 1.41E-4x +0.03282	0.998	0.005-10	0.005
Trp	Trp-IS	y = 1.09E-3x -0.11358	0.997	0.005-10	0.005
Glu	Glu-IS	y = 4.31E-5x +0.00279	0.998	0.005-10	0.005
Val	Val-IS	y = 5.91E-5x +0.00035	0.999	0.005-10	0.005
Phe	Phe-IS	y = 8.00E-5x -0.00284	0.999	0.005-10	0.005
Leu	Leu-IS	y = 5.31E-4x -0.00401	0.998	0.005-10	0.005
Ser	Ser-IS	y = 6.72E-5x +0.04392	0.998	0.005-10	0.005
Thr	Thr-IS	y = 5.37E-5x +0.01625	0.999	0.005-10	0.005
Ala	Ala-IS	y = 3.05E-5x +0.00386	0.999	0.005-10	0.005
His	His-IS	y = 3.89E-4x -0.01020	0.999	0.005-10	0.005
Lys	Lys-IS	y = 9.85E-5x +0.00123	0.999	0.005-10	0.005
Pro	Pro-IS	y = 7.92E-5x -0.01116	0.999	0.005-10	0.005
Met	Met-IS	y = 6.47E-5x -0.07170	0.999	0.005-10	0.005
Asn	Asn-IS	y = 2.86E-6x -0.00129	0.999	0.010-10	0.010
Gly	Gly-IS	y = 3.38E-5x +0.01118	0.999	0.005-10	0.005
Ile	Ile-IS	y = 9.04E-5x +0.00825	0.999	0.010-10	0.010
Cys	Cys-IS	y = 1.41E-4x -0.05073	0.991	0.010-10	0.010
Tyr	Tyr-IS	y = 1.68E-4x -0.02539	0.999	0.005-10	0.005

Table S3 The calibration curves and the LOQ of the tryptophan metabolites

Compound	Internal standard	Linearity	R^2	Linear Range （μmol/L）	LOQ （μmol/L）
HK	serotonin-IS	y = 0.1933x +0.0074	0.997	0.010-5	0.010
KA	KA-IS	y = 0.6067x +0.1887	0.995	0.005-5	0.005
AA	AA-IS	y = 0.0018x -0.0023	0.973	0.005-5	0.005
QA	serotonin-IS	y = 0.0245x +0.1117	0.996	0.020-5	0.020
KYN	KYN-IS	y = 0.2369x +0.0194	0.999	0.005-5	0.005
HIAA	HIAA-IS	y = 0.2546x +0.0610	0.996	0.005-5	0.005
XA	Tryptophan-IS	y = 0.0050x +0.00001	0.994	0.005-5	0.005
ILA	AA-IS	y = 0.0144x +0.0057	0.996	0.010-5	0.010
HIAA	HIAA-IS	y = 4.6032x +0.2249	0.996	0.010-5	0.010
IAA	IAA-IS	y = 0.3041x +0.0627	0.995	0.005-5	0.005
IPA	IAA-IS	y = 0.5038x +0.0855	0.998	0.005-5	0.005
Serotonin	serotonin-IS	y = 1.0422x +0.0126	0.998	0.005-5	0.005
Tryptamine	Tryptamine-IS	y = 159.27x +14.465	0.998	0.002-5	0.002
Tryptophan	Tryptophan-IS	y = 0.8643x +0.2406	0.998	0.005-5	0.005
Melatonin	Melatonin-IS	y = 1.9558x -0.0293	0.998	0.005-5	0.005

Table S4 List of MRM parameters of amino acids and their internal standards[a]

Compounds	Q1 mass	Q3 mass	DP	CE	CXP	polarity
Ala	218.1	130.1	59	17	10	positive
[U-^{13}C,U-^{15}N]-Ala	222.1	133.1	59	17	10	positive
Asn	243.1	157.1	16	15	6	positive
[U-^{13}C,U-^{15}N]-Asn	249.1	163.1	16	15	6	positive
Cys	497.2	248.1	89	23	6	positive
[U-^{13}C,U-^{15}N]-Cys	505.2	252.1	89	23	6	positive
Gln	275.2	172.1	61	19	10	positive
[U-^{13}C,U-^{15}N]-Gln	282.1	178.1	61	19	10	positive
Glu	318.2	230.1	64	18	12	positive
[U-^{13}C,U-^{15}N]-Glu	324.2	235.1	64	18	12	positive
Gly	204.1	144.1	56	12	12	positive
[U-^{13}C,U-^{15}N]-Gly	207.1	147.1	56	12	12	positive
His	370.2	196.1	26	29	10	positive
[U-^{13}C,U-^{15}N]-His	379.2	204.1	26	29	10	positive
Ile	260.2	130.1	53	25	10	positive
[U-^{13}C,U-^{15}N]-Ile	267.2	136.1	53	25	10	positive
Leu	260.2	172.1	58	17	10	positive
[U-^{13}C,U-^{15}N]-Leu	267.2	178.1	58	17	10	positive
Lys	361.2	301.2	71	14	8	positive
[U-^{13}C,U-^{15}N]-Lys	369.2	309.2	71	14	8	positive
Met	278.1	190.1	16	15	10	positive
[U-^{13}C,U-^{15}N]-Met	284.1	195.1	16	15	10	positive
Phe	294.2	206.1	60	16	11	positive
[U-^{13}C,U-^{15}N]-Phe	304.2	215.1	60	16	11	positive
Pro	244.2	184.1	50	12	10	positive
[U-^{13}C,U-^{15}N]-Pro	250.2	190.1	50	12	10	positive
Ser	234.1	174.1	50	14	10	positive
[U-^{13}C,U-^{15}N]-Ser	238.1	178.1	50	14	10	positive
Thr	248.1	160.1	53	16	8	positive
[U-^{13}C,U-^{15}N]-Thr	253.1	164.1	53	16	8	positive
Trp	333.2	245.1	68	22	10	positive
[$^{2}H_5$]-Trp	338.2	250.1	68	22	10	positive
Tyr	396.2	308.2	84	19	9	positive
[U-^{13}C,U-^{15}N]-Tyr	406.2	317.2	84	19	9	positive
Val	246.2	158.1	58	16	13	positive
[U-^{13}C,U-^{15}N]-Val	252.2	163.1	58	16	13	positive

[a] DP, Declustering potential; CE,Collision energy; CXP, Cell exit potential

Table S5 List of MRM parameters of tryptophan metabolites and its internal standards [a]

Compounds	Q1 mass	Q3 mass	DP	CE	CXP	polarity
QA	168	149.9	41	15	10	positive
QA-IS	171	153	41	15	10	positive
HK	225.2	208	41	13	10	positive
HK-IS	228.2	211	41	13	10	positive
Serotonin	177.2	160.1	41	15	8	positive
Serotonin-IS	181.1	164	41	15	8	positive
KYN	209.1	192.0	40	13	8	positive
KYN-IS	213.1	196	40	13	8	positive
HAA	154.2	80	46	36	6	positive
HAA-IS	156.2	82	46	36	6	positive
Tryptophan	205.1	118.1	39	26	6	positive
Tryptophan-IS	210.2	122.0	39	26	6	positive
Tryptamine	161	144	36	15	11	positive
Tryptamine-IS	165	148	36	15	11	positive
XA	206.2	160	56	27	11	positive
KA	190.2	144.0	51	30	6	positive
KA-IS	195.2	149	51	30	6	positive
HIAA	192.2	146	31	21	6	positive
HIAA-IS	197.2	151	31	21	6	positive
AA	138.2	120	36	15	10	positive
AA-IS	142.1	124	36	15	10	positive
ILA	206.2	130	36	39	10	positive
Melatonin	233.1	174	56	21	12	positive
Melatonin-IS	237.1	178	56	21	12	positive
IAA	176.2	130	56	25	12	positive
IAA-IS	178.2	132	56	25	12	positive
IPA	190.2	130.1	51	22	12	positive

[a] DP, Declustering potential; CE,Collision energy; CXP, Cell exit potential

Table S6 The gradient of UPLC-MS/MS for amino acids separation

Time(min)	Flow rate (μL/min)	H_2O (0.1% formic acid)	Acetonitrile (0.1% formic acid)
0	300	40	60
12	300	20	80
12.01	300	2	98
15	300	2	98
15.01	300	40	60
22	300	40	60

Table S7 The gradient of UPLC-MS/MS for tryptophan metabolites separation

Time(min)	Flow rate (μL/min)	H_2O (0.1% formic acid)	Acetonitrile (0.1% formic acid)
0	300	100	0
10	300	60	40
12	300	5	95
12.01	300	100	0
17	300	100	0

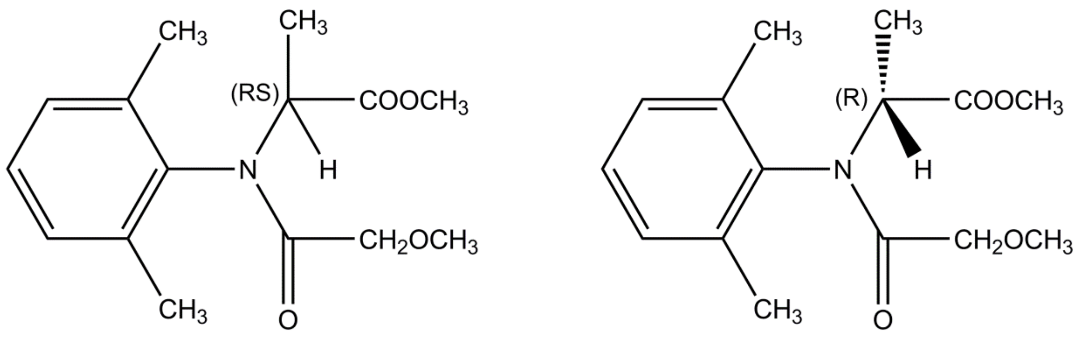

rac-metalaxyl   metalaxyl-M

Figure S1. Chemical structures of *rac*-metalaxyl and metalaxyl-M

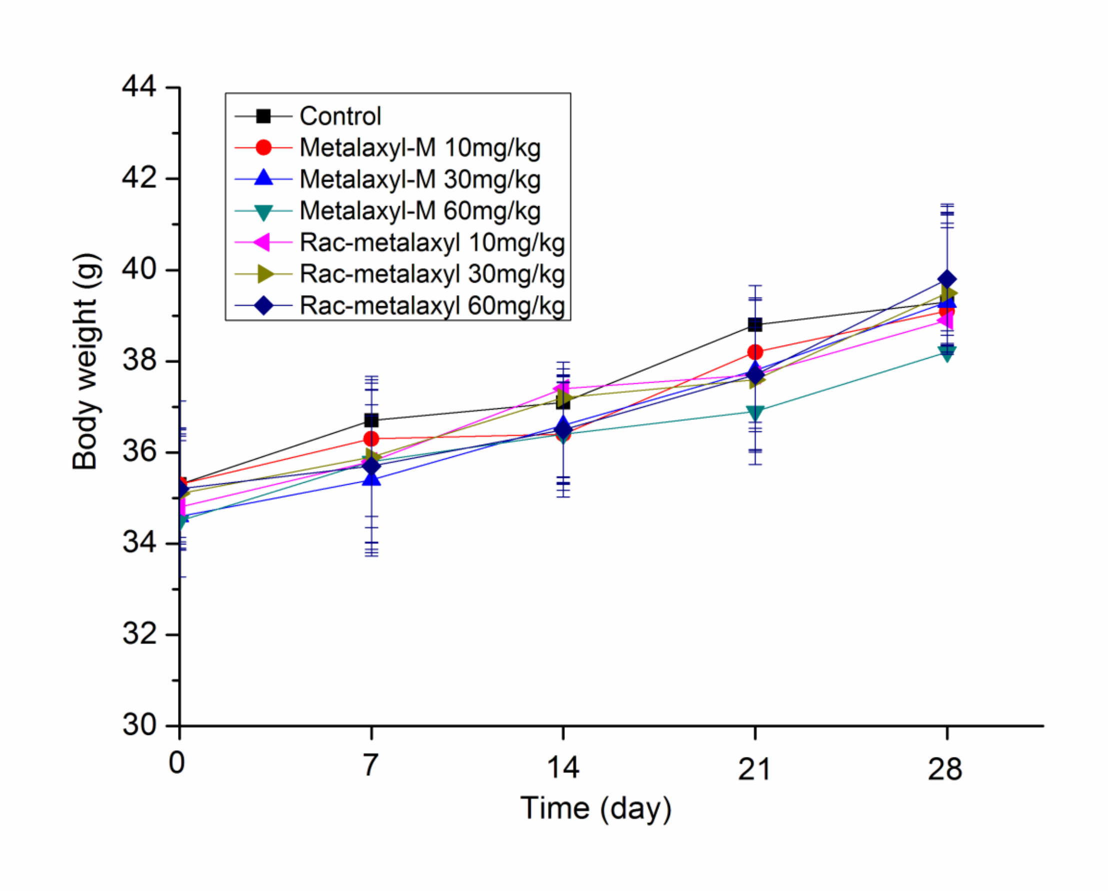

Figure. S2 Body weight changes of mice after *rac*-metalaxyl and metalaxyl-M exposure

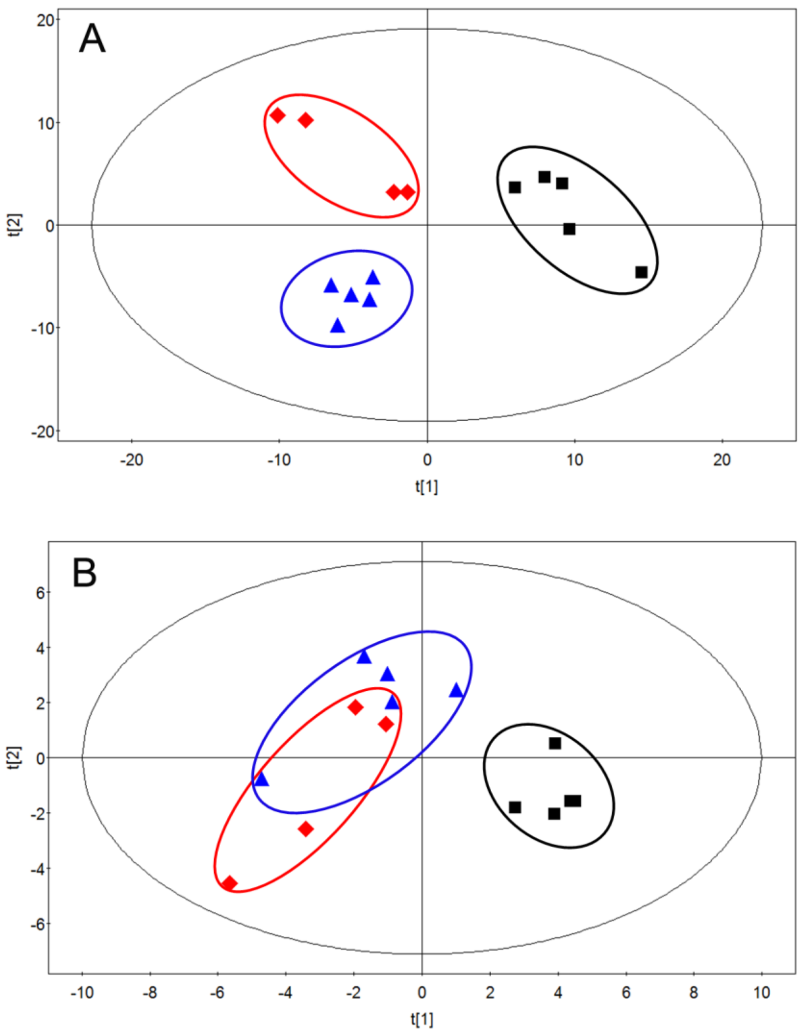

Figure S3 Partial least-squares discriminant analysis (PLS-DA) based on urine ^{1}H-NMR spectra. (A) metalaxyl-M (R2X = 0.724, R2Y = 0.857, Q2 = 0.932); (B) *rac*-metalaxyl(R2X = 0.615, R2Y = 0.71, Q2 = 0.824); (■) control group, (▲) 30 mg/kg treated group (◆) 60 mg/kg treated group

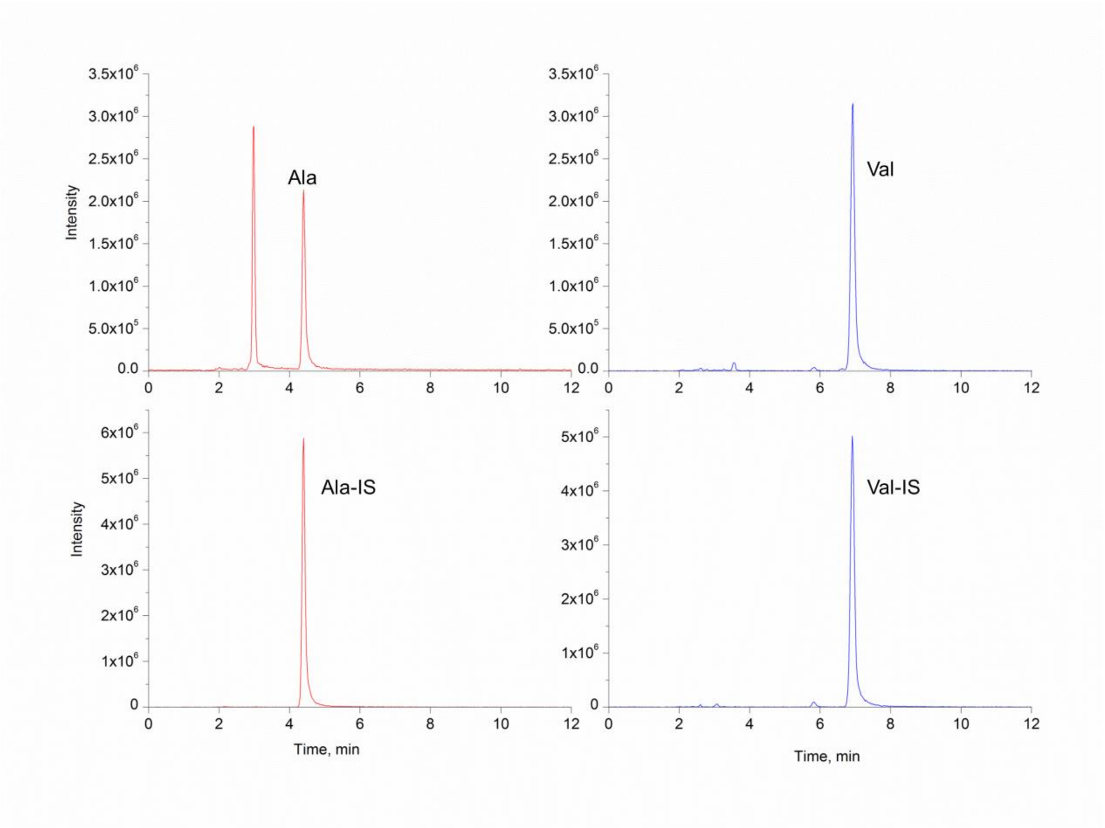

Figure S4 Representative LC-MS/MS chromatograms of alanine, valine and their stable isotope labeled internal standards.

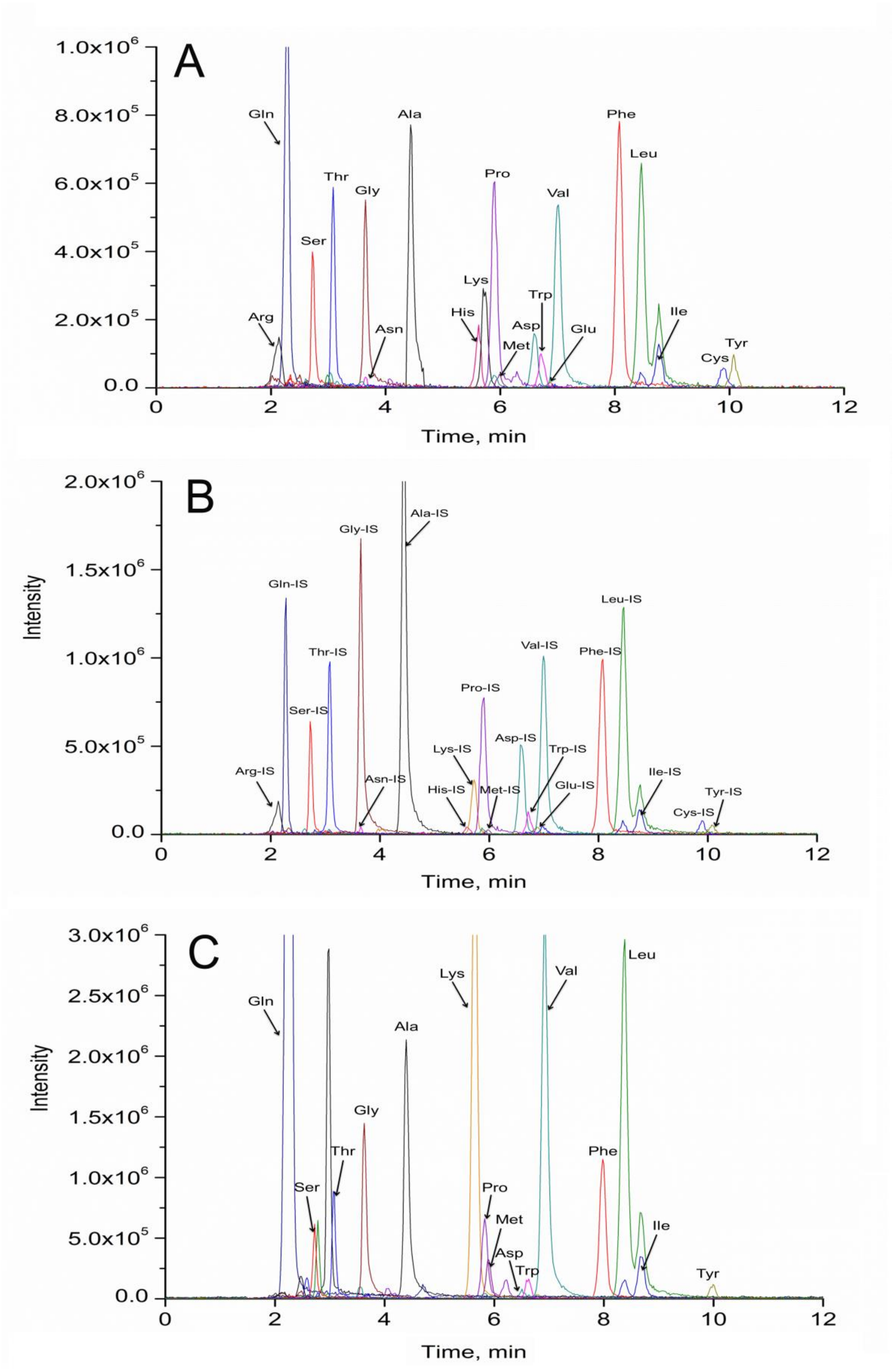

Figure S5 Representative LC-MS/MS chromatograms of amino acids. (A) unlabeled amino acids metabolites; (B) isotope labeled internal standards; (C) serum sample
